# Behavioral and Neurobiological Convergence of Odor, Mood and Emotion: A Review

**DOI:** 10.3389/fnbeh.2020.00035

**Published:** 2020-03-10

**Authors:** Ioannis Kontaris, Brett S. East, Donald A. Wilson

**Affiliations:** ^1^Givaudan UK Limited, Health and Well-being Centre of Excellence, Ashford, United Kingdom; ^2^Emotional Brain Institute, Nathan Kline Institute for Psychiatric Research, Orangeburg, NC, United States; ^3^Child and Adolescent Psychiatry, NYU School of Medicine, New York University, New York, NY, United States

**Keywords:** odor, odor perception, emotion, fear, mood, fragrance, memory

## Abstract

The affective state is the combination of emotion and mood, with mood reflecting a running average of sequential emotional events together with an underlying internal affective state. There is now extensive evidence that odors can overtly or subliminally modulate mood and emotion. Relying primarily on neurobiological literature, here we review what is known about how odors can affect emotions/moods and how emotions/moods may affect odor perception. We take the approach that form can provide insight into function by reviewing major brain regions and neural circuits underlying emotion and mood, and then reviewing the olfactory pathway in the context of that emotion/mood network. We highlight the extensive neuroanatomical opportunities for odor-emotion/mood convergence, as well as functional data demonstrating reciprocal interactions between these processes. Finally, we explore how the odor- emotion/mood interplay is, or could be, used in medical and/or commercial applications.

## Introduction

It is commonly held that odors, even when presented without conscious awareness, can modulate emotion and mood (Herz, 2009; Kadohisa, 2013). For example, 5 min of exposure to an unpleasant odor (e.g., pyridine) has been reported to induce a negative mood and mild anxiety, while 5 min of exposure to a pleasant odor (e.g., commercial perfume) can induce positive mood and calming (Villemure et al., 2003). However, the terms *emotion* and *mood* have overlapping, though distinct, psychological definitions and increasingly are believed to have distinct neurobiological underpinnings. Do odors have a similar impact on both moods and emotions? In this manuscript, we briefly review the psychology and biology of mood and emotion in humans and then highlight links between olfaction, mood and emotion at the neural circuit and behavioral levels based on data derived from both humans and animal models. The focus is not on whether odors can directly evoke emotions or have hedonic valence—they do and this may be a fundamental adaptation of the olfactory system (Herz, 2000; Yeshurun and Sobel, 2010). Rather the focus is on how odors may modulate ongoing emotional or mood states. Finally, we explore how the odor-mood/emotion interplay is, or could be, used in commercial and medical applications.

## Definitions and Caveats

We begin our brief overview of the vast and active field of research on emotion and moods with basic definitions necessary to help align neuroscientists and psychologists. For more in-depth definitions and discussions of these terms see reviews by LeDoux (1996), Russell (2003) and Barrett et al. (2007). The term *emotion* [or core affect (Russell, 2003)] generally refers to the immediate response to the anticipation or occurrence of rewarding or punishing stimuli or events. Emotions thus tend to be short-lived and event- or stimulus-driven and have a valence (i.e., good or bad). Classic basic (“natural”) emotions include happiness, sadness, anger, fear, surprise, and disgust. In humans, emotions are subjective evaluations of underlying physiological and behavioral responses to threat or reward (LeDoux, 2014). Thus, seeing a snake can trigger a number of physiological and behavioral responses to deal with the potential threat, e.g., activation of the sympathetic nervous system to mobilize energy for escape leading to rapid heart rate and respiration, activation of skeletal muscle system to move the body away from the threat, or in some cases just the opposite—behavioral freezing. Such physiological and behavioral reactions can be observed in both humans and animal models. However, in humans, overlying these physiological and behavioral responses is the conscious subjective interpretation of what one’s body is doing; resulting, in this case, in the emotion of fear (Russell, 2003; LeDoux and Pine, 2016). A different set of physiological and conscious responses may be evoked upon the sight of one’s favorite dessert or true love. An emotional experience (compared to a non-emotional experience) entails the coherent organization of all these components (Russell, 2003; Delplanque et al., 2017).

Given the inability to divine conscious, subjective, experiences in non-human animal models, there is debate over whether non-human animals display classic emotions in the full sense just described. This review article will not settle that debate. Nonetheless, our understanding of the neurobiology of circuits underlying the physiology of such behaviors is well informed by non-human animal research. As noted by Barrett et al. (2007): “animal models yield necessary and important insights that must be incorporated into any model of emotion, but they have not (and probably cannot) give a sufficient account of the events people call fear, anger, or sadness” (page 298). Thus, our discussion of the neurobiology of olfaction, emotions and moods below relies on both human and non-human animal data.

**Figure 1:**
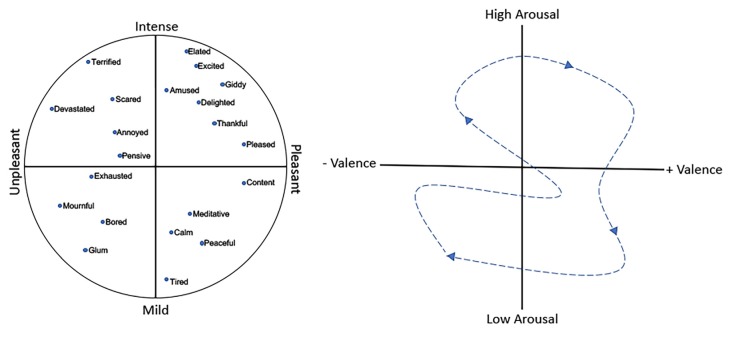
(Left) An example of a two-dimensional emotion plot including intensity and valence dimensions. (Right) A representation of the temporal shift in the affective state (dashed arrow) which reflects the combination of emotion and mood. Over time, an individual expresses an emotional/mood trajectory through different states.

Furthermore, there is some debate over whether there are specific, discrete emotions, e.g., fear, anger, love, or whether emotions fall along continuous dimensions (Panskepp, 1998; Mendl et al., 2010; Hamann, 2012; Lindquist et al., 2013). A variety of models have been developed to describe those dimensions (Russell, 2003; Coppin and Sander, 2016), though most include a valence dimension (i.e., pleasant vs. unpleasant or reward vs. punishment) and an arousal dimension (i.e., high vs. low or intense vs. mild), with the third dimension of potency sometimes also included. Figure 1 shows an example of a two-dimensional emotion plot. In this plot, fear represents relatively high arousal, negative valence emotion, as opposed to excited, which is also relatively high arousal but has a positive valence. On the arousal dimension, both excited and peaceful have a similarly positive valence but differ along the intensity dimension. In support of this dimension gradient hypothesis, neuroimaging data (discussed below) suggests that rather than discrete emotions each associated with activity in specific brain regions, emotions and their underlying neuroanatomy may fall along a gradient, with no finite boundaries between circuits associated with one emotion and another (Hamann, 2012; Lindquist et al., 2012).

Metaphorically, emotions are to moods, as the weather is to climate. In contrast to emotions, moods [or prolonged core affect (Russell, 2003)] are relatively long-lasting (Trimmer et al., 2013). Furthermore, moods are not necessarily induced by specific events or stimuli but can be internally generated. Moods reciprocally interact with emotions. For example, a series of events evoking negative emotions can induce a negative mood. Similarly, being in a particular mood state can influence the valence or intensity of a sensory-evoked emotion. For example, being in a negative mood may reduce the strength of a positive emotional response to a favored food. In the extreme, moods can be very extended and pathological, such as depression or mania.

Thus, at any given moment an individual’s affective state is the combination of emotion and mood, with mood reflecting the running average of sequential emotional events together with an underlying internal affective state. Over time, assuming no psychopathology, an individual expresses an emotional/mood trajectory through different states (Figure 1). The question is how can olfactory stimuli influence this trajectory? Odors have intrinsic hedonic valence themselves (Mandairon et al., 2009; Yeshurun and Sobel, 2010; Perry et al., 2016) and can acquire or modify their hedonic valence through experience (Cain and Johnson, 1978; Mennella and Garcia, 2000; Delplanque et al., 2017). However, odors have many other perceptual properties that could drive emotion/mood toward higher or lower arousal states, as well as along the valence dimension (Chen et al., 2006; Albrecht et al., 2011; Trellakis et al., 2012; Cecchetto et al., 2019). Furthermore, such an odor-induced influence on the trajectory could potentially occur without conscious awareness of the odor’s presence (Chen et al., 2006). What neural circuits may allow an odor-evoked activity to impact emotion and mood circuits? The following section reviews current understanding of neural circuits mediating emotion and mood, as derived from human and non-human animal data, as well as circuits underlying odor perception, in order to highlight potential critical points of convergence.

## Neurobiology

In order to explore how odors may modulate emotion and mood, we first review circuits believed to be important for each of these three phenomena. Identifying the neural substrates and circuits underlying specific behaviors is notoriously difficult, even when the behavior involved is relatively simple and well-defined. For example, the brain regions and circuits involved when a person picks up a pencil seen lying on a table—a seemingly simple, clear movement behavior—include retina, thalamus, brainstem, and visual cortical areas necessary for locating the pencil, which in turn project to motor planning and control regions to guide arm movement. In addition, somatosensory feedback is required for grasping the pencil, which involves an additional thalamocortical circuit. Depending on the distance and location of the pencil, vestibular and balance circuits may need to be active to keep from falling over during the reach. In addition, to these components, the network is heavily modulated by diverse inputs providing information about the internal and external context that could directly influence visual perception and/or movement control. Finally, the conscious perception of those stimuli (e.g., awareness that one sees a visual stimulus categorized as a pencil) involves regions downstream from the primary sensorimotor regions (Romo and Rossi-Pool, 2020). Together, this rich, complex network is involved in a very simple, precisely-defined sensorimotor behavior.

Compared to picking up a pencil, emotions can be far more complex. Understanding the neural circuits underlying the complex and more fluidly-defined phenomena of emotion and mood, thus, raises challenges. Furthermore, for reasons of both medical translational importance and relative ease of experimental control, most work in this field focuses on fearful emotions and anxious or depressive moods (Rauch et al., 2006; Ressler and Mayberg, 2007; Shin and Liberzon, 2010; LeDoux and Pine, 2016), although increasing efforts have been made to examine happiness, reward, and positive emotions, which may be involved in addictive behaviors (Burgdorf and Panksepp, 2006; Kringelbach and Berridge, 2009). Together, substantial progress in emotional network analyses has been made. Here, we describe basic circuits associated with stimulus/event-induced emotions and/or their underlying physiological states, followed by a survey of mood-associated brain activity.

### Neural Circuits and Emotion

Assuming that emotional responses lie along a continuum rather than existing as discrete, labeled-line sensorimotor behaviors (Mendl et al., 2010), it is unlikely that specific brain regions are uniquely and selectively involved in specific emotions (Hamann, 2012; Lindquist et al., 2012). For example, research regarding the emotional functions of the various amygdala nuclei is overwhelmingly dominated by fear, anxiety, and the response to threat (LeDoux, 1996), however, the amygdala is also involved in encoding positive valenced stimuli, as well as simple arousal induced by stimuli of any valence in both humans and non-human animals (Lang and Davis, 2006; Bonnet et al., 2015; Janak and Tye, 2015; Jin et al., 2015; Weymar and Schwabe, 2016).

Rather, much like the distributed sensorimotor circuits underlying specific sensory-guided movements described above, circuits controlling emotional encoding and responses (e.g., autonomic, skeletal, and cognitive) also appear to be highly distributed. Thus, different emotions may rely on networks whose components are shared with other networks depending on emotional valence or intensity (Tye, 2018). There may be some specialization of network components, for example, circuits coupled closely with autonomic reflexes or to specific sensory inputs. However, the distributed, overlapping nature of the controlling networks favors gradients of emotional expression over highly discrete, 1-region:1:emotion configurations, though this is still an area of very active debate (Kober et al., 2008; Vytal and Hamann, 2010; Hamann, 2012; Lindquist et al., 2012, 2013).

There are several lines of evidence to support the distributed network view of emotion in both humans and animal models. Imaging studies in humans have used two different approaches to delineate emotional neural networks. The first and most common method involves using discrete emotion labels to analyze neural data in search of an associated network (e.g., Vytal and Hamann, 2010). For example, one might examine activity levels in specific regions of interest and network connectivity when the subjects were, by some operational definition, in a specific emotional state. This method, of course, begins with the assumptions that there are specific, basic emotions and that those emotions can be accurately categorized and induced. The second method analyses neural networks without applying assumed, discrete, emotion labels. In this procedure the question becomes, for example, in a population of subjects expressing a variety of emotions, are there a variety of stable, selective networks which can be identified and could then be used to classify emotion (e.g., Kober et al., 2008)? This latter approach avoids the conflict of imposing what may be artificial, emotional constructs/classifications onto emergent networks (Kober et al., 2008).

Using these different approaches and definitions, several consistent emotion-related brain regions and neural networks [recently labeled the “affectome” (Becker et al., 2019)] have been identified. It must be emphasized at the outset, however, that even well-identified brain regions are generally very heterogeneous structures, in terms of both cell types and function. For example, manipulations of small regions of the nucleus accumbens in rodents allow mapping of sub-regional hotspots with an activity that drives appetitive (e.g., food-seeking—positive hedonic) behavior and neighboring regions less than 1–2 mm away with activity drives fearful (e.g., defensive—negative hedonic) behavior (Berridge and Kringelbach, 2015). Importantly for the purposes of this review, environmental context affects this map. That is, while rodents are in their quiet home cage, surrounded by their nest, food, and familiar smells, the population of neurons within the nucleus accumbens driving appetitive behaviors expands. In contrast, when they are in a well-lit, noisy, unfamiliar environment, the defensive zone expands (Reynolds and Berridge, 2008). Thus, neurons change which circuits they are involved in (appetitive/defensive), depending on the state and situation of the animal. Therefore, as noted above, suggesting that region × is always critical for emotional function Y is generally, at best, an over-simplification.

**Figure 2:**
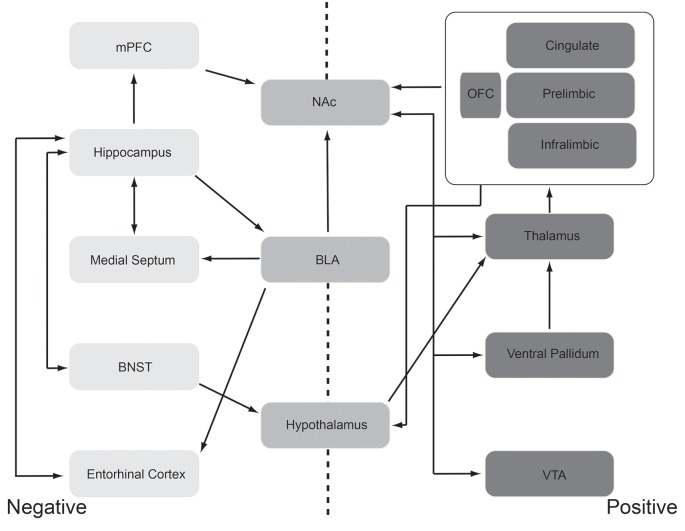
Schematic representation of the “affectome” (Becker et al., 2019), i.e., a network of mood/emotion-related brain regions. This schematic should be interpreted with the caveat that even well-identified, mood/emotion-related brain regions are generally very heterogeneous structures both structurally and functionally. Regions most commonly aligned with negative emotions (e.g., aversion, anxiety, fear learning, etc.) are shown lightly shaded and to the left and those associated with positive emotions (e.g., attraction, reward) are shown darkly shaded to the right. Functionally heterogeneous regions are shown in the center. These regions have been identified in both human and animal model research as being important for the expression emotional responses, and in some cases the recognition of emotional cues in others, through a variety of techniques including activity suppression/lesions, activation, electrophysiology, neuroanatomy, and functional imaging. See text for a discussion of support for this network. Abbreviations: BLA, the basolateral nucleus of the amygdala; BNST, bed nucleus of the stria terminalis; mPFC, medial prefrontal cortex; NAc, nucleus accumbens; OFC, orbitofrontal cortex; VTA, ventral tegmental area.

Given those caveats, circuits involved in the expression and recognition of emotional states have been identified (Figure 2). In both humans and animal models, circuits aligned with positive emotions (e.g., pleasant, liking responses) are relatively distinct from, though overlap extensively with, circuits aligned with negative emotions (e.g., defensive, aversive responses). For example, the brain regions and circuits are shown on the right in Figure 2 are most strongly, consistently associated with positive emotions. These regions include orbitofrontal, insular and anterior cingulate cortices, nucleus accumbens, ventral pallidum, amygdala, lateral hypothalamus, ventral tegmental area, and the parabrachial nucleus. This circuit thus ranges from the brainstem to highest order neocortex. Each of these regions has been identified as involved in positive emotions through either direct manipulation (primarily in animal models) or monitoring activity (humans and animal models). For example, regions within the orbitofrontal cortex (OFC) modulate their activity in accordance with the rewarding nature or pleasantness of stimuli and modify that response as the rewarding value changes with habituation or changes in state. Thus, a hungry animal may have a strong orbitofrontal response to a food odor, but with more feedings, the pleasantness/reward value of that odor decreases, as does the response in the OFC (O’Doherty et al., 2000).

Furthermore, as noted above, each region is not necessarily a homogeneous functional unit. As shown in animal models, in many areas, sub-regional emotional hot-spots exist, wherein modulation of one hotspot’s activity may drive positive affect (e.g., enhanced sucrose preference), while manipulating a neighboring spot within the same brain region may suppress sucrose preference or even induce sucrose disgust responses (Berridge and Kringelbach, 2015). Thus, a given brain region may lie within both the positive and negative emotion networks depending on the state and recent history.

That said, Figure 2 schematically highlights the primary regions most commonly aligned with negative emotions (e.g., aversion, anxiety, fear learning, etc.) and with positive emotions (e.g., attraction, reward). These regions have been identified in both human and animal model research as being important for the expression of emotional responses, and in some cases the recognition of emotional cues in others, through a variety of techniques including lesions, electrophysiology, neuroanatomy, and functional imaging. There is an extensive literature supporting these networks and network components and their role in emotion that will not be fully reviewed here. Rather, we present these networks as a basis for our exploration of how odors can influence emotion. Are there points of convergence between the olfactory and emotion circuits that could support olfactory modulation of emotion? This will be the focus of “Olfactory Modulation” section.

### Neural Circuits and Mood

In contrast to the relatively transient nature of emotions, moods are typically considered to be an extended state, though similar states can be expressed as either emotions or moods. However, it should be noted that the concept of mood, or prolonged effective state, “is fuzzy because neither duration nor degree of stability is defined” (page 147; Russell, 2003). For example, anxiety can be either a transient emotional states or a prolonged mood. Thus, much of the circuitry described in the previous section for emotion may also hold for moods. However, while brief anxiety in animal models may be associated with elevated amygdala activation and relieved by amygdala suppression (Tye et al., 2011), an anxious mood can be associated with relatively stable changes in neurogenesis, brain region size, levels of neuromodulator release, hypothalamic-pituitary-adrenal axis dysregulation, and changes in autonomic nervous system tone (Anacker and Hen, 2017). Similarly, while there may be similarities in basic circuits underlying a brief period of sad emotion and major depressive disorder, the latter can involve changes in prefrontal cortical activity in both patients and animal models (Post and Warden, 2018), and serotonergic, dopaminergic and glutamatergic neurochemistry, neuroinflammation, and function of the hypothalamic-pituitary-adrenal axis in animals (Post and Warden, 2018; Cathomas et al., 2019). This suggests that, while circuits may be similar, the efficacy of odors to modulate emotions and moods may differ. An odor that relieves a sad emotion following a favorite sports team’s loss, may have much less impact on the major depressive disorder, though this difference has not been explicitly examined in the research literature.

### Neural Circuits and Odor Perception

Olfaction plays a critical role in food localization and identification (i.e., energy balance), identification of threats, mates and kin, and finding home in most vertebrates and invertebrates (Wyatt, 2017). Food odors can evoke cephalic-phase digestive system responses as if food had actually been consumed (e.g., salivation), body odors can be used to guide social preferences (Li et al., 2007) and can serve as social buffers during stress in humans and animal models (Sullivan and Toubas, 1998), even in the absence of the individual, and odors can evoke relaxation or anxiety/fear dependent on their past associations. For example, odors associated with battle can evoke anxiety, fear, flight, and/or panic responses in individuals with PTSD (Daniels and Vermetten, 2016) while maternal odors can reduce crying in human infants (Sullivan and Toubas, 1998). Thus, given the role of odor cues in behavior, it should be clear that odors and emotion, and potentially mood, are closely aligned. What are the neuroanatomical bases for this alignment? Here, we provide an overview of the functional anatomy of the olfactory system. Detailed reviews of local circuit anatomy and function are published elsewhere (Cleland and Linster, 2003).

Odor perception, like emotional and cognitive phenomena, is a neural network-based process. This network includes hierarchical and parallel upstream pathways, as well as extensive top-down and lateral connectivity. The mammalian olfactory pathway is an evolutionarily old pathway which does not align with other canonical, thalamo-neocortical sensory pathways. Importantly, for the purposes of this review, primary olfactory regions have direct, often reciprocal connections with many of the regions involved in emotion and mood outlined above in both humans and animal models.

Olfactory sensation and perception begin with olfactory sensory neurons in the nose. These receptor cells are true neurons with cilia that extend into the olfactory mucous and are dense with olfactory receptor proteins. Sensory neuron output is *via* axons that directly enter the forebrain and synapses within the olfactory bulb. Most olfactory receptor proteins preferentially bind specific chemical moieties of volatile ligands. Thus, rather than a receptor that binds jasmine, the percept of jasmine begins with a chemical analysis of the diverse molecules within the jasmine odor source, with individual receptors recognizing submolecular features (e.g., hydrocarbon chains of different length, functional groups such as esters and aldehydes, etc.). That feature information is transmitted to the olfactory bulb where it evokes a unique, odor-specific, spatial-temporal pattern of activity in second-order neurons as well as mitral and tufted cells. Extensive inhibitory networks within the olfactory bulb enhance the contrast between patterns, as well as modulate odor-driven activity in a state- and context-dependent manner. For example, the rodent olfactory bulb response to food odor is strongly modulated by hunger and satiety (Palouzier-Paulignan et al., 2012). This top-down input may contribute to the recent findings of odor hedonic valence being encoded in the olfactory bulb of both rodents (Kermen et al., 2016) and humans (Iravani et al., in preparation), or that information may be extracted from olfactory sensory neuron input directly by the bulb.

It should be noted that in addition to this feature-driven, odor-object process, there is also evidence for “labeled-line”-like odor-evoked behaviors. For example, in many non-human animals, volatile molecules that bind to specific receptors evoke specific behaviors such as sexual attraction (Wyatt, 2017). These chemical cues are called pheromones and can elicit activity in both the main and accessory (vomeronasal) olfactory systems. However, recent evidence suggests that activation of some select sensory neurons in the main olfactory system may also drive approach/avoidance behaviors directly (Dewan et al., 2013; Saito et al., 2017; Horio et al., 2019). Given that it is unlikely that activation of these receptors occurs in isolation of activation of many other receptors (e.g., when inhaling a natural mixture), these “labeled-line” pathways could convey innate preferences/aversions to odor objects.

Output neurons of the olfactory bulb, which receive direct input from olfactory sensory neurons, then project that information to a wide range of anatomically and functionally diverse targets in both humans (Fjaeldstad et al., 2017) and non-human mammals (Cleland and Linster, 2003). The direct targets of olfactory bulb output include anterior olfactory nucleus (AON), olfactory tubercle (OT), cortical nucleus of the amygdala (coA), anterior and posterior piriform cortex [PCX; anterior piriform cortex (aPCX) and posterior piriform cortex (pPCX) respectively], and lateral entorhinal cortex (LEC; Cleland and Linster, 2003). Each of these structures plays a unique role in translating chemical-evoked activity in the periphery into odor perception.

For example, based on animal models, the AON serves as the major gateway for bilateral interaction between the two olfactory bulbs (Brunjes et al., 2005) which may be important for odor localization (Kikuta et al., 2010; Esquivelzeta Rabell et al., 2017) and is involved in odor feature convergence (Lei et al., 2006). However, more recently in mice, it has also been shown to be a site of early convergence between episodic memory generated in the hippocampus and odor information *via* a direct hippocampal-AON pathway (Aqrabawi and Kim, 2018). Interestingly, in rodents, AON neurons are also directly responsive to oxytocin, a hormone involved in a variety of physiological and cognitive/emotion functions including milk release during lactation, orgasms, and social affiliative behaviors (Churchland and Winkielman, 2012). In rodents. elevated oxytocin, *via* its action in the AON, can enhance recognition of social odor cues, such as those of partners or kin (Oettl et al., 2016). In humans, oxytocin also modulates responsiveness to social odors (Maier et al., 2019), though a link to receptors in AON has not been determined.

The olfactory tubercle is a component of both the olfactory cortex and the ventral striatum (Wesson and Wilson, 2011; Xiong and Wesson, 2016) and has been described in both humans (Sobel et al., 2000) and animal models (Wesson and Wilson, 2011). As olfactory cortex, it receives direct input from the olfactory bulb, though unlike other olfactory cortical areas, it does not send a return projection to the bulb (In’t Zandt et al., 2019). It is heavily interconnected with sensory, cognitive, hormonal, and reward-related regions of the brain (Xiong and Wesson, 2016) and maybe involved in attention to odors (Carlson et al., 2018) and odor-induced motivation (Murata et al., 2015), as might be expected given its strong link to dopaminergic reward and motor circuits (Ikemoto, 2007). The rodents OT is also monosynaptically connected with the amygdala and hypothalamus. Together, this connectivity pattern places OT as an interface between odors and emotion, motivation and cognition in rodents and potentially humans as well. Furthermore, given that many of these connections are reciprocal, the OT allows emotion, motivation, and cognition to modulate odor responses.

Several nuclei of the amygdala can receive olfactory input including the medial, basolateral, and the coA, and activity of the amygdala closely track odor valence in humans (Jin et al., 2015; Sorokowska et al., 2016) and animal models (Root et al., 2014; Perry et al., 2016; Iurilli and Datta, 2017). In rodents, the anterior coA receives direct input from the olfactory bulb, as well as from the aPCX and pPCX (Pitkanen, 2000; Cleland and Linster, 2003). Recent work in rodents suggests that the coA may be especially involved in innate, aversive/attractive responses to odors and less involved in learned odor hedonic responses (Root et al., 2014; Iurilli and Datta, 2017). The coA, in turn, projects to the basolateral amygdala (BLA), among other targets (Pitkanen, 2000; Majak et al., 2004; Thompson et al., 2008). The BLA also has a strong, reciprocal connection with the pPCX (Majak et al., 2004). Thus, as with the OT, the coA allows a close direct connection with regions involved with emotion and memory, and this relationship is bidirectional. For example, optogenetic activation of BLA modulates pPCX single-unit responses to odor (Sadrian and Wilson, 2015). This would suggest that how odors are processed, and thus perceived, can be shaped by amygdala activity (i.e., emotional state) in the same way that emotional state may be modulated by odor. In fact, in humans, exposure to affective-inducing pictures modifies odor thresholds and pleasantness ratings (Pollatos et al., 2007).

The largest component of the olfactory cortex is the PCX, which can be divided into at least two major components, the aPCX and pPCX in both humans (Howard et al., 2009) and animal models (Haberly, 2001). While both regions receive direct input from the olfactory bulb, they differ in (1) neuronal subpopulations (Large et al., 2018), (2) the nature and/or density of projections they receive from other brain regions (Majak et al., 2004), and (3) their projections to other brain regions (Chen et al., 2014; Diodato et al., 2016). Activity in the PCX most closely correlates with, and/or predicts, odor perception in humans (Gottfried, 2010) and rodents (Kadohisa and Wilson, 2006). It has been hypothesized that the PCX merges odorant features extracted by the receptors and olfactory bulb into perceptual odor objects (Haberly, 2001; Wilson and Sullivan, 2011), providing for both odor quality coding (e.g., rose) and odor categorization (e.g., floral; Kadohisa and Wilson, 2006; Howard et al., 2009). The PCX performs this odor object construction in an experience-dependent manner and is critically involved in odor memory (Calu et al., 2007; Sacco and Sacchetti, 2010; Meissner-Bernard et al., 2019; Terral et al., 2019; Zhang et al., 2019).

As with the other olfactory regions, the PCX is closely, reciprocally connected to a variety of regions involved in emotion and mood including the BLA, OFC, perirhinal cortex, mediodorsal nucleus of the thalamus, and lateral hypothalamus based on animal model anatomy (Cleland and Linster, 2003; Wang et al., 2020) and human functional connectivity data (Fjaeldstad et al., 2017). Thus, in addition to being critical for the encoding of odor objects and remembering them, the PCX can serve as an important, bi-directional interface between odors and emotions/moods. In support of this interface role, recent work has shown that odors differing in hedonic valence can evoke both different patterns of activity within, and different network functional connectivity between, PCX and other brain regions (Krusemark et al., 2013; Kondoh et al., 2016; Perry et al., 2016).

Finally, mammalian olfaction is dependent on breathing. Recent work in humans has demonstrated that respiration, which is known to entrain neural activity throughout the olfactory pathway, also entrains olfactory system activity with activity in emotion- and memory-relevant regions, such as amygdala and hippocampus (Zelano et al., 2016; Heck et al., 2019). Changes in respiration, such as voluntary slow breathing during relaxation techniques, can modulate odor-driven memory recall (Masaoka et al., 2012). In fact, the emotion itself can modulate respiration, in part, *via* connections with brainstem respiratory centers (Del Negro et al., 2018). This suggests that emotion-driven changes in respiration could shape odor memory and perception. Conversely, odor inhalation can also modify respiration volume and/or rate (Mainland and Sobel, 2006), potentially providing an alternative pathway for odor-driven modulation of emotion network activity.

## Olfactory Modulation

### Behavioral Links Between Odor, Mood, and Emotion

#### Mood

Previous literature has shown how pleasant and unpleasant smells can have a powerful effect on mood. For example, a series of studies have used the Profile of Mood States (POMS) and Total Mood Disturbance (TMD; Schiffman et al., 1995a) to assess the effect of cologne use on mood. The POMS has been shown to detect transient mood shifts and assess mood across six factors: tension-anxiety, depression-dejection, anger-hostility, vigor-activity, fatigue-inertia, and confusion-bewilderment. As assessed with these measures, the use of colognes as part of a daily routine for male and female participants enhanced ratings of mood at midlife (Schiffman et al., 1995b,c). Conversely, compared to controls, people living close to intensive swine operations reported reductions in mood ratings from the emanating unpleasant smells (Schiffman et al., 1995a).

Given that humans spend 90% of their time indoors, indoor air quality can be an important factor for mood and health (Brasche and Bischof, 2005). Reports of stuffy or dry air are common occurrences in domestic and work environments. Previous studies have found that perceived air pollution in indoor environments is influenced by human body odors, building materials, ventilation systems, indoor smoking, and other human activities (Fanger et al., 1988). One study found that cleaning, an activity closely associated with pleasant odors, was a significant factor in reduced symptoms of Sick Building Syndrome (SBS) compared to no-odor conditions (Wang et al., 2013).

In addition to odors that are strong enough to be consciously perceived, weak, potentially subconscious, ambient odors have been shown to influence mood states in a positive or negative manner (Kirk-Smith et al., 1983; Zucco et al., 2009), as well as intentions and actual food choices (Gaillet et al., 2013; Gaillet-Torrent et al., 2014), buying intentions (Glatzel, 2010), and cleaning behavior (Holland et al., 2005).

To sum up, several reports have shown that pleasant odors result in an increase in self-reported mood whereas unpleasant odors result in the reverse effect. In other words, the valence dimension of odors features heavily in affecting mood in a manner comparable to other manipulations and seems to be necessary, but not sufficient, to induce mood enhancement (Lehrner et al., 2000; Retiveau et al., 2004; Delplanque et al., 2017). The effect of odors on moods are critically dependent on past associations and personal preferences for those odors (Herz, 2009). It is important to note that the effects on putative mood states of odor are occasionally assessed relative to a no-odor control, rather than as a comparison between odors (Lehrner et al., 2000; Wang et al., 2013), thus interpreting the efficacy of a given odor relative to others, which could provide insight into neural mechanisms, remains unclear.

#### Emotion

Over the last 10 years the scientific literature has witnessed a dramatic increase in interest in emotional measurements and, as a result, has examined numerous manifestations of the mood and emotion experience, such as cognitive, facial, motor/somatosensory, psychophysiological, and neural components. These investigations are typically preceded by an introduction that highlights the differences between moods and emotions (e.g., with respect to their differences in duration). However, following this, the terms mood and emotion are used interchangeably, sometimes without taking care to ensure the terminology reflects the measures and experimental design is used. This blurring of boundaries between the psychological constructs of emotion and mood, in turn, blurs the search for underlying neural circuits. Understanding the neurobiology of behavior requires, as a starting point, a precise, quantifiable metric of the behavior.

With this important caveat aside, evidence shows that odors can modulate ongoing emotional states. For example, research by Knasko (1992, 1995) demonstrated that the smell of baby powder, chocolate, or lavender resulted in reports of more positive emotional state, whereas dimethyl sulfide had the opposite effect. More recently, bread and cucumber odors increased mood but did not change choice behavior in a more realistic, less controlled setting of a buffet restaurant (Mors et al., 2018).

Furthermore, studies employing physiological and brain measures, such as heart rate, galvanic skin response, electroencephalography (EEG), or functional magnetic resonance imaging (fMRI) have shown the ability of odors to elicit relaxation or invigoration and relief from anxiety or stress. For instance, Diego et al. (1998) found that lavender oil promoted relaxation and decreased anxiety with a concurrent increase of alpha power in EEG. Conversely, induction of anxiety can change the perception of neutral odors to aversive, and modify activity and connectivity with the olfactory cortex and its connected partner regions (Krusemark et al., 2013).

Interestingly, odors can have effects on emotions and behavior even when presented without the subject consciously perceiving them, similar to the mood state effects described above. However, this effect is context-dependent, influenced by individual factors and does not hold true for all odors. For instance, Lundström and Olsson showed that subliminal levels of androstadienone odor enhanced women’s mood compared to control participants who had not been exposed to the odor (Lundström and Olsson, 2005). However, this effect was only significant when an experimenter of the opposite sex was in the room. Hence emotional and behavioral modulation was enabled by the social context during odor exposure. More recently, in an fMRI study, participants were exposed to a fragrance or body odors concealed by the same fragrance and were asked to choose between deontological or utilitarian actions in different types of real and fake dilemmas (Cecchetto et al., 2019). The authors found that the body odors masked by fragrance could increase the emotional experience during the decision-making process and thereby influence moral choices.

### Neural Circuit Links Between Odor, Mood, and Emotion

**Figure 3:**
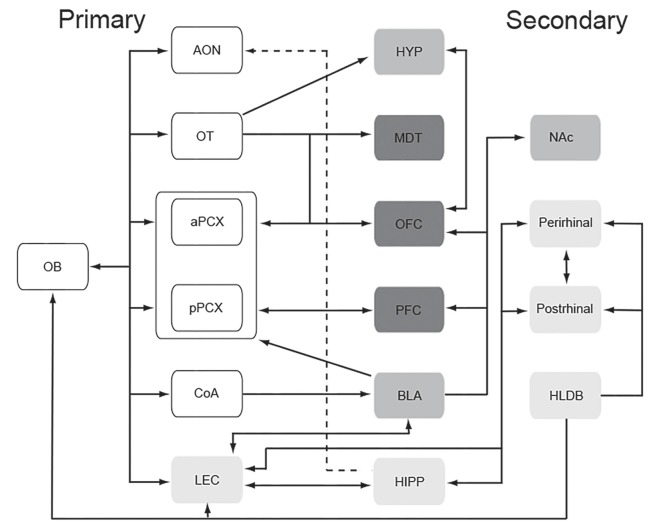
Schematic representation of the primary (direct input from the olfactory bulb) and secondary (one synapse beyond primary regions) olfactory network. Shading identifies the same regions as shown in the affectome (Figure 2). This remarkably tight, often reciprocal relationship between early stages of the olfactory system and the connectome may promote odor modulation of mood and emotion. Abbreviations: AON, anterior olfactory nucleus; aPCX, anterior piriform cortex; BLA, basolateral amygdala; CoA, cortical nucleus of the amygdala; HIPP, hippocampus; HLDB, horizontal limb of the diagonal band; HYP, hypothalamus; LEC, lateral entorhinal cortex; MDT, mediodorsal thalamus; NAc, nucleus accumbens; OB, olfactory bulb; OFC, orbitofrontal cortex; OT, olfactory tubercle; PFC, prefrontal cortex; pPCX, posterior piriform cortex.

As described above, the olfactory system and circuits involved in emotion and mood heavily overlap and interconnect (Soudry et al., 2011). Both portions of olfactory cortex itself (e.g., coA, OT, LEC) and regions one synapse beyond the primary olfactory pathway (e.g., OFC, perirhinal cortex, BLA, insular cortex, hippocampus, lateral hypothalamus, BNST) are regions typically identified as involved in emotion and mood (compare Figures 2, 3). This tight, generally reciprocal relationship between olfactory and emotion/mood circuits is reflected in the fact that, as also described above, odors can affect emotions and mood, and emotions and mood can influence odor perception. In this section, we briefly describe a specific example to underscore this relationship.

Anxiety is an emotional state characterized by apprehension and threat avoidance in the absence of threat. The amygdala plays a major role in anxiety, and suppression of BLA output, especially to the central nucleus of the amygdala, can be anxiolytic (Tye et al., 2011). Brief induction of anxiety in humans can induce a change in odor perception such that normally neutral odors come to be perceived as unpleasant, and odor sensitivity can be modified. This anxiety-induced change in odor perception is associated with enhanced PCX and OFC odor-evoked activity, and enhanced functional connectivity between the amygdala and olfactory cortex (Krusemark et al., 2013). Mood-related changes in neural connectivity and odor responses could, in part, be induced through anxiety-related changes in cortisol (Hoenen et al., 2017) or through direct changes in neural activity.

Conversely, odor exposure can affect anxiety (Ballanger et al., 2019). In both humans and animal models, odors including lavender (Chioca et al., 2013), citrus scents (e.g., *Citrus sinensis* essential oil, linalool; Lehrner et al., 2000), and green leaf odors (e.g., 2-hexenal; Nakatomi et al., 2008) can have anxiolytic effects, in some cases *via* circuits and signaling cascades, comparable to those induced by pharmacological treatments. For example, in animal models, lavender aroma exposure may modify GABA receptor signaling (Ballanger et al., 2019) or serotonergic signaling (Chioca et al., 2013), both of which are targeted by some pharmacological anxiolytics. Similarly, green leaf odor that can produce anti-depressive effects in mice acts in part *via* elevation of serotonin, similar to some pharmacological anti-depressants such as selective serotonin reuptake inhibitors (Nakatomi et al., 2008). However, as noted above many of these studies rely exclusively on a no-odor control, and thus do not allow direct comparisons of the efficacy of different odors to induce these effects.

## Applications

### Medical Applications

As described here and elsewhere (Herz, 2009; Ballanger et al., 2019), the evidence for olfactory modulation of mood and emotion is increasingly strong, and the neural networks underlying mood, emotion, and olfaction are tightly interwoven, which helps support an odor-mood/emotion interaction. That said, there are many unsubstantiated claims about the therapeutic effects of odors, and an unfortunate common theme of aromatherapy research is under-powered design with limited experimental control (Herz, 2009). Furthermore, any long-term odor treatment would require addressing issues related to receptor adaptation and central habituation, which would be expected to reduce the efficacy of odor exposure over prolonged or repeated presentations. Perceptual habituation may not, however, eliminate odor effects on mood and emotion given the effects of weak or subconscious presentations outlined above.

Nonetheless, there are examples of basic and clinical research that are providing promising support for an olfactory role in the treatment and/or amelioration of mood/emotion disorders. The most ideal application would be to target mood disorders (e.g., depression, anxiety) rather than short-term emotions, though in some situations even short-term emotions could be a useful target of odor modulation. Here, we outline four potential medical applications of odor-modulated mood/emotion that have research support: anxiety, depression, emotional disturbance associated with ageing and dementia, and insomnia. In each case, we very briefly describe some supporting evidence and outline caveats that must be addressed before such treatments could be widely applied.

#### Anxiety

Some odors can be anxiolytic (Chioca et al., 2013; Krusemark et al., 2013; Ballanger et al., 2019), though odorant efficacy may be expected to vary substantially between individuals, and even within individuals, over time or in different contexts. Furthermore, the olfactory system is very effective at habituating to background odors (Wilson and Linster, 2008; Pellegrino et al., 2017). Long-term treatment of anxiety with odor exposure would require addressing receptor adaptation and central habituation, which would be expected to reduce efficacy. Treating or preventing more acute anxiolysis, such as anxiety induced by a visit to the dentist or when taking an exam, maybe more tractable (Lehrner et al., 2000), though again, interpersonal variables will be an issue to be addressed. Furthermore, anxiety and stress itself can modify olfactory function (Krusemark et al., 2013; Hoenen et al., 2017), which could interact with any planned therapeutic effect.

#### Depression

In humans, there is a strong, reciprocal relationship between impaired olfaction and depression. Thus, subjects with depression have reduced olfactory ability and patients with olfactory impairment show a positive correlation between the severity of smell loss and symptoms of depression (Kohli et al., 2016). However, odors may be efficacious in treating some aspects of depression. For example, there is some evidence in animal models that odors can reduce depressive behaviors (Nakatomi et al., 2008). Note, however, that depression is associated with reduced olfactory abilities, especially after prolonged depression, and that loss of smell itself induces depression (Kohli et al., 2016; Pabel et al., 2018). Thus, the same caveats apply here as mentioned with anxiety treatment.

#### Ageing and Dementia

Ageing (Doty and Kamath, 2014; Seubert et al., 2017) and dementia, especially Alzheimer’s disease (Murphy, 1999; Devanand et al., 2015) and Parkinson’s disease (Doty, 2012) are associated with olfactory loss. This severely impacts using odors to manipulate mood and emotion in the aged. Nonetheless, some work has explored using odor to ameliorate cognitive and emotional deficits in this population. For example, dementia is associated not only with cognitive impairment but often also with emotional dysregulation and anxiety (Gulpers et al., 2016; Isaacowitz et al., 2017). There is emerging evidence that odors may help reduce anxiety in ageing (Ballanger et al., 2019), though much more work is required to clarify these potential effects.

Sleep plays a critical role in emotional regulation and memory consolidation (Yoo et al., 2007; Altena et al., 2016), and sleep is often impaired in normal and pathological ageing (Mander et al., 2017). Although odor detection is greatly reduced during NREM sleep in humans (Carskadon and Herz, 2004) and animal models (Barnes et al., 2011), odors presented during NREM sleep in humans modulate respiration (Arzi et al., 2010) and enhance NREM delta power (Perl et al., 2016), which could enhance sleep quality. Finally, sensory stimulation, including odor training, can improve sensory function in the elderly, which in turn can enhance cognitive functioning (Birte-Antina et al., 2018; Leon and Woo, 2018).

### Commercial Applications

Differentiation of modern products is becoming more difficult since technical performance and hedonic properties operate at a high level. In addition, the rate of failures in the marketplace indicates liking measures are not reliable predictors of success. Emotional and behavioral characteristics of products are becoming more and more important to establish a unique selling proposition at the product and brand level.

The fragrance is a key driver during purchase decisions for cosmetic and consumer products. The fragrance in the product can be designed to elicit specific memories (Sugiyama et al., 2015), moods and emotions (e.g., Behan et al., 2004; Kontaris et al., 2018) during crucial moments, such as during smelling on-shelf, the decision to purchase, and during different stages of use. Furthermore, the fragrance is part of a whole product equation that includes consumer experience and expectation from top-down effects, with brain responses to contextual effects and hedonic valence in the olfactory system visible as early as the olfactory bulb in both humans (Iravani et al., in preparation) and animal models (Freeman and Schneider, 1982; Mandairon and Linster, 2009; Kermen et al., 2016). Hence, products can be designed holistically by integrating the right fragrance in the right packaging design and ensuring appropriate odor-color-emotion correspondences and apposite emotional effects. This kind of optimization is akin to a musical orchestra where all elements, including the instruments, the acoustics, the environment, and the level of skill contribute to the product experience. The right balance can lead to a delightful consumer reaction, making the product or service memorable, as well as standing out at the point of purchase and beyond.

Indeed, at a time when failure rates for most new products are high (Thomson and Crocker, 2015), products and brands that create an emotional connection with consumers are more likely to be a success in the market (Hollins and Pugh, 1990). To this effect, the consumer products and consumer fragrance and flavors industries need to build solid investigations on how to measure emotional and behavioral effects of fragrance and the mechanisms behind such interactions.

A challenge unique to smell is that people have difficulty in communicating aspects of their smell experiences verbally (Engen, 1982). A common approach in the literature has been to generate pre-determined or consumer-led lexicons for use in verbal self-report. However, classical verbal measures such as questionnaires often fail to capture the true spectrum of emotional experience (Porcherot et al., 2016), are more prone to demand characteristics and variations in the labels that individuals assign to emotions (Quirin et al., 2009), and are more susceptible to halo effects from hedonics (e.g., Nisbett and Wilson, 1977). Additionally, if participants are oblivious to these emotional states (Jostmann et al., 2005), such measures may not be appropriate to utilize.

To this effect, researchers have previously used non-verbal and implicit techniques to assess the emotional effects of fragrance. For example, one of the authors (IK) has used proprietary emotional measurements such as Mood Portraits^®^ (Churchill and Behan, 2010), which overcomes the need for verbal responses by inviting consumers to select pre-screened pictures they feel best to represent their current mood after smelling a fragrance. This capability has been applied globally and elucidated the emotional effects of cosmetics, fragrance, and flavored products in several countries around the world (Kontaris et al., unpublished data). One advantage of these types of techniques is they require less cognitive load. In addition, previous work has shown that pictures are superior to words in terms of speed of processing (Seifert, 1997; Azizian et al., 2006; Hinojosa et al., 2009) and allow more direct access to meaning, privileged access to emotional information (Dehouwer and Hermans, 1994), and stronger and more widespread brain activity (Kensinger and Schacter, 2006).

These findings and techniques are routinely used by the business and perfumery teams when briefed to develop fragrances and flavors which emotionally benefit consumers and create a specific mood ambience in different parts of the world. Importantly, quantification of emotional effects can differentiate equally liked products on a number of dimensions (e.g., Ng et al., 2013). Lastly, data analytics of such findings can be used to create perfumery guidelines for the intelligent design of fragrances with specific emotional benefits to consumers of different product categories in different parts of the world (e.g., Behan et al., 2004; Kontaris et al., 2018).

### Going Forward

Olfaction has been driving approach and avoidance behaviors throughout evolutionary time and has even been hypothesized to be synonymous with emotion (Herz, 2000; Yeshurun and Sobel, 2010). As outlined above, the neuroanatomy of the olfactory system and circuits underlying mood and emotion are closely linked in both humans and non-human animal models, and there is increasingly strong evidence that odors can both evoke hedonic responses and influence mood and/or emotion. However, there are a variety of unanswered questions and a need for greater rigor in research on this topic. The excellent review by Herz 10 years ago (Herz, 2009) on aromatherapy, which included odor effects on mood, outlined both the strengths and weaknesses of research in this field. Many of the weaknesses remain, including the need for: (1) greater sampling size and statistical power; (2) greater stimulus control; and (3) greater awareness of the effects of context and individual differences which shape odor effects (e.g., age, gender, genetics, cultural background, odor experience, and preferences).

We add to this list the need to distinguish between effects on emotion and mood. For example, it is not clear that experimentally-induced, transient anxiety (i.e., a brief emotional state) models a prolonged anxiety mood disorder. Given that the neurobiology of transient anxiety and anxiety mood disorder differ, so might the efficacy of odor manipulations. The same holds true for depression and other mood disorders. Thus, in exploring the effects of odors on these phenomena, there must be some attention paid to emotion and mood differences.

In addition, it remains the case that humans are not rodents and vice versa. Emotions and moods are conscious interpretations of internal states that may, or may not have true homologs in non-human animal models. For example, despite decades of study on the neurobiology of “fear” in rodent models through training of rodents to “freeze” in response to cues that signal an aversive stimulus, “mechanisms that detect and respond to threats are not the same as those that give rise to conscious fear” (LeDoux, 2014; page 2871). Furthermore, a rodent may display depression-like behavior that is treatable with anti-depressants, but whether the rodent is depressed in the way a human would experience is unknown. Similarly, while there are many strong parallels between circuits underlying human and non-human animal odor processing, much of the precise anatomical connectivity between olfactory and emotion/mood circuits is not known in humans. For example, while functions and connections of the anterior and posterior PCX appear to align closely between humans (Howard et al., 2009) and rodents (Kadohisa and Wilson, 2006), definitions of the precise location and size of the human olfactory tubercle still vary widely (Wesson and Wilson, 2011). The point here is not to discount translation between animal models and humans. Remarkable progress has been made in understanding neurobiological mechanisms of perception, cognition and behavior through animal models. Rather, it is critical to remain aware of the potential limitations of those models, especially in an area where the conscious experience of emotion and mood is so critical to the outcome being measured.

Finally, olfactory deficits are associated with many pathologies, including schizophrenia, depression, and many forms of dementia. This association reflects the strong links between olfaction and emotion/mood networks and raises the possibility that loss of olfactory function may serve as an early biomarker of many disorders, and/or serve as a potential avenue for treatment of at least some symptoms. However, reduced and/or altered odor perception in the same population one wishes to treat with odors is an obvious problem. One option is that olfactory exposure and training can improve olfactory abilities and sensitivity (Wysocki et al., 1989; Hummel et al., 2009; Al Aïn et al., 2019; Fleming et al., 2019), which could potentially re-open the window to odor modulation of mood and emotion.

## Author Contributions

IK, BE and DW contributed to the writing of this review manuscript and figure preparation.

## Conflict of Interest

IK was employed by the company Givaudan UK Limited.
